# Correction: Autophagy protein 5 controls flow-dependent endothelial functions

**DOI:** 10.1007/s00018-023-04916-3

**Published:** 2023-09-04

**Authors:** Pierre Nivoit, Thomas Mathivet, Junxi Wu, Yann Salemkour, Devanarayanan Siva Sankar, Véronique Baudrie, Jennifer Bourreau, Anne-Laure Guihot, Emilie Vessieres, Mathilde Lemitre, Cinzia Bocca, Jérémie Teillon, Morgane Le Gall, Anna Chipont, Estelle Robidel, Neeraj Dhaun, Eric Camerer, Pascal Reynier, Etienne Roux, Thierry Couffinhal, Patrick W. F. Hadoke, Jean-Sébastien Silvestre, Xavier Guillonneau, Philippe Bonnin, Daniel Henrion, Joern Dengjel, Pierre-Louis Tharaux, Olivia Lenoir

**Affiliations:** 1 Inserm, Université Paris Cité, PARCC, 56 Rue Leblanc, 75015 Paris, France; 2 Centre for Cardiovascular Science, The Queen’s Medical Research Institute, University of Edinburgh, Edinburgh, EH16 4TJ UK; 3 Department of Biomedical Engineering, University of Strathclyde, Glasgow, G4 ONW UK; 4 Department of Biology, University of Fribourg, 1700 Fribourg, Switzerland; 5 MITOVASC, CNRS UMR 6015, Inserm U1083, Université d’Angers, 49500 Angers, France; 6 Département de Biochimie et Biologie Moléculaire, Centre Hospitalier Universitaire d’Angers, 49500 Angers, France; 7 CNRS, Inserm, Bordeaux Imaging Center, BIC, UMS 3420, US 4, Université de Bordeaux, 33000 Bordeaux, France; 8 Plateforme Protéomique 3P5-Proteom’IC, Institut Cochin, INSERM U1016, CNRS UMR8104, Université Paris Cité, 75014 Paris, France; 9 Inserm, Biologie Des Maladies Cardiovasculaires, U1034, Université de Bordeaux, 33600 Pessac, France; 10 Institut de La Vision, INSERM, CNRS, Sorbonne Université, 75012 Paris, France; 11 AP-HP, Hôpital Lariboisière, Physiologie Clinique - Explorations Fonctionnelles, Hypertension Unit, Université Paris Cité, 75010 Paris, France

**Correction: Cellular and Molecular Life Sciences (2023) 80:210** 10.1007/s00018-023-04859-9

In this article, authors Pierre-Louis Tharaux and Olivia Lenoir have contribute equally to this work.

The original article has been updated.

